# Consultative meeting to develop a strategy for treatment of cutaneous leishmaniasis. Institute Pasteur, Paris. 13–15 June, 2006

**DOI:** 10.1186/1475-9292-6-3

**Published:** 2007-04-24

**Authors:** Farrokh Modabber, Pierre A Buffet, Els Torreele, Geneviéve Milon, Simon L Croft

**Affiliations:** 1 Drugs for Neglected Diseases Initiative (DNDi), 1 Place St Gervais, Geneva CH 1201, Switzerland; 2 Center for Research and Training in Skin Diseases and Leprosy, Tehran University of Medical Sciences, 79 Taleghani Avenue, Tehran 14166, Iran; 3 Centre Médical de l'Institut Pasteur, 28 rue du Docteur Roux, Cedex 15, Paris 75724, France; 4 Dept Parasitology and Mycology, Institut Pasteur, 25 Rue du Docteur Roux, Cedex 15, Paris 75724, France

## Abstract

**Background:**

A meeting was organized by Drugs for Neglected Diseases initiative (DNDi) and the Institute Pasteur (IP), Paris, to review the treatment for all forms of cutaneous leishmaniasis (CL) and to propose a strategy for the development of new efficacious and affordable treatments.

**Method:**

The global burden of CL was discussed with respect to financial impact; relation to poverty; the stigma of CL lesions and scars (particularly in young women); lack of effective, affordable, easily implemented tools and political will and resources to implement available control tools; and lack of input from pharmaceutical and biotechnology companies to develop new drugs and vaccines.

**Results:**

According to the experts from different endemic countries present, the financial and social burdens of CL are high, but we have limited quantitative data. The analysis of published trials indicates that the quality of most trials is poor and requires both improvement and standardization. The available drugs are inadequate. Criteria by which different CL types could be prioritized as target disease were set. These criteria included: severity of the disease; lack of response to available drugs; overall incidence and prevalence of the disease; sequelae of the disease, (including recidivans and mucosal leishmaniasis); the impact of treatment of individuals on control of transmission and lack of other major parties involved in drug development. Based on these, the anthroponotic CL and its sequel "recidivans" caused by *L. tropica *and CL caused by *L. braziliensis *and its sequel, mucosal leishmaniasis were considered to be the target diseases.

The mechanism for controlling *Leishmania *infection to reach a stable self healing process is a balanced immune response. Immune stimulation during chemotherapy can enhance cure. There is no adequately effective vaccine, but some encouraging results have been obtained with whole killed *Leishmania *parasites or imiquimod (an immuno-modulator) plus antimonials. Further studies are needed. One safety/immunogenicity clinical trial is currently ongoing with a Second Generation Vaccine (SGV).

**Conclusions and recommendations:**

There is an urgent need for new treatments for all CL types. CL should be considered as a neglected disease and organizations, such as DNDi, should include it in their list of target diseases. It was agreed that immuno-chemotherapy (with "therapeutic" vaccines or immunomodulators) has a strong potential to make an impact as a new therapy of CL with the view of shortening/reducing duration and dose of drug treatment and preventing resistance. There is also a need for safe, affordable and efficacious new chemotherapeutics. The quality of clinical trials needs to be enhanced and standardized. Short and long-term objectives and activities were defined as a part of meeting recommendations.

## Review

**Figure 1:**
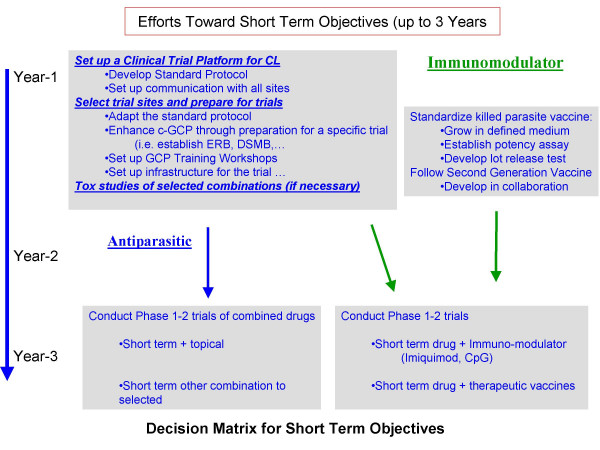
**Schematic representation of decision matrix for short-term objectives (3 years)**. ctivities related to trials of selected combination of anti-parasitic drugs will proceed in parallel with development of immuno-chemotherapy approach using available drugs, immun-modulators, with or without leishmanial antigens. Efforts will be focused on enhancing the quality of clinical trials through development of standard protocols and ICH-GCP training.

**Figure 2:**
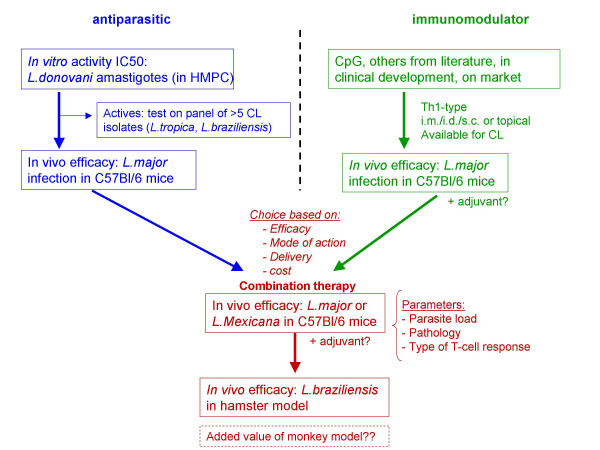
**Schematic representation of decision matrix for long-term objectives (7–10 years)**. Two parallel activities similar to those in short-term objectives is envisaged. New potential drug identified through synthesis/screening against *L. donovani *will be tested against isolates of *Leishmania *causing CL in a mouse model. Active compounds will be identified for further development and eventually tested in combined immuno-chemotherapy trials. New immuno-modulators will be tested in the B6 mouse model; however, the CpG's with human-specific motifs being developed for cancer therapy will be adopted for CL treatment.

A meeting, organized by the Drugs for Neglected Diseases initiative (DNDi) and IP, was held to review the present situation of treatment of all forms of CL, to elaborate on the socio-economic impact of this disease on individuals and the financial burden to endemic countries, to identify gaps in knowledge and tools and to recommend alternative models for developing new safe, efficacious and affordable treatments. Invited participants were experts actively engaged in research and treatment of cutaneous leishmaniasis, representing experience in over 14 countries (see Table 1 for list of participants). The meeting followed immediately after another organized by IP, Pasteur Institute Tunis and the Walter Reed Army Institute of Research (WRAIR) on a new topical treatment in clinical development for CL caused by *L. major *so that latest progress could be shared. All participants submitted a brief overview of their presentation, distributed to all prior to the meeting. Short presentations were followed by sessions allotted to brain-storming and discussions. Following the first afternoon of presentations, participants were divided into two groups to discuss short term (3–5 years) and long term (up to 10 years) objectives and priorities, and to develop decision matrices for these objectives (Fig. 1 &2). Participants considered CL and its sequels that encompass many different disease entities with different epidemiology, causative *Leishmania spp*., natural history, public health importance, the role of case treatment in the control of disease, the response to available treatment, and new and required tools for treatment. The need to identify the target diseases, to develop a target product profile (TPP, See Appendix 1 ACL-TPP.xls) and a set of recommendations by consensus were agreed.

**Table 1:** List of Participants

**Last Name**	**First Name**	**City**	**Country**	**email**
Alvar	Jorge	Geneve	Switzerland	alvarj@who.int
Arana	Byron	Guatemala City	Guatemala	baaz@cdc.gov
Ben Salah	Afif	Tunis	Tunesia	Afif.bensalah@pasteur.rns.tn
Bogdan	Christian	Freiburg	Germany	christian.bogdan@uniklinik-freiburg.de
Buffet	Pierre	Paris	France	pabuffet@pasteur.fr
Croft	Simon L.	Geneva	Switzerland	s.croft@dndi.org
Don	Robert	Geneva	Switzerland	rdon@dndi.org
Garnier	Tracy	Herts	UK	t.garnier@herts.ac.uk
Ghalib	Hashim	Geneva 27	Switzerland	ghalibh@who.int
Grogl	Max	Hawaii	USA	max.grogl@haw.tamc.amedd.army.mil
Hailu	Asrat	Addis	Ethiopia	hailu_a2004@yahoo.com
Hommel	Marcel	Paris	France	mhommel@pasteur.fr
Khamesipour	Ali	Tehran	Iran	Khamesipour_ali@yahoo.com
Lang	Thierry	Paris Cedex	France	tlang@pasteur.fr
Lecoeur	Hervé	Paris	France	helecoe@pasteur.fr
Llanos-Cuentas	Alejandro	SMP Lima	Peru	allanos@upch.edu.pe
Magill	Alan	Washington DC	USA	alan.magill@na.amedd.army.mill
Matlashewski	Greg	Montreal, Quebec	Canada	greg.matlashewski@mcgill.ca
Meaume	Sylvie	Ivry sur Seine	France	Sylvie.meaume@cfx.aphp.fr
Meheus	Filip	Antwerp	Belgium	filip.meheus@ua.ac.be
Milon	Geneviève	Paris Cedex 15	France	gmilon@pasteur.fr
Modabber	Farrokh	Ferney-Voltaire	France	modabberf@yahoo.com
Morizot	Gloria	Paris	France	gmorizot@pasteur.fr
Pecoul	Bernard	Geneva	Switzerland	bpecoul@dndi.org
Rabello	Ana	Belo Horizonte	Brazil	ana@cpqrr.fiocruz.br
Rizzo	Nidia	Guatemala City	Guatemala	nrrz@cdc.gov
Royce	Catherine	Geneva	Switzerland	croyce@dndi.org
Sacks	David	Bethesda	USA	dsacks@nih.gov
Stahl	Kurt-Wilhelm	Freiburg	Germany	kwstahl@web.de
Torreele	Els	Geneva	Switzerland	etorreele@dndi.org
**Unable to attend**				
Boelaert	Marleen	Antwerpen	Belgium	mboelaert@itg.be
Brewer	Thomas	Seattle, WA	USA	Thomas.brewer@gatesfoundation.org
Davis	Heather	Ottawa, Ontario	Canada	hdavis@coleypharma.com
Khalil	Eltahir A.G.	Khartoum	Sudan	eltahirk@iend.org
Olliaro	Piero	Geneva	Switzerland	oliarop@who.int
Reed	Steven G.	Seattle, WA	USA	sreed@idri.org

To begin, Ms. M. Boccoz, Director of International Relations of IP welcomed the guests and as IP is a founding partner of DNDi, was pleased that the meeting was being held at the Institute which looked forward to further collaboration and participation in the global fight against "Neglected Diseases" with DNDi. Dr Croft, Director, R&D DNDi also welcomed the participants on behalf of DNDi, and outlined the goals and activities to: (a) Develop new drugs for people suffering from neglected diseases (b) Ensure equitable access to new and field-relevant health tools, (c) Raise awareness of the need to develop drugs for neglected diseases and (d) build public responsibility and leadership in addressing needs of these patients. DNDi was established in July 2003 as a virtual model, working with institutions around the world to meet the specific needs of most neglected diseases. DNDi has developed partnerships with institutes in disease-endemic countries and counts on their involvements. Currently DNDi has over 20 projects in 2006 portfolio, from discovery to clinical trials on human African trypanosomiasis, visceral leishmaniasis, cutaneous leishmaniasis, Chagas disease and malaria. The future focus will be on the trypanosomiases and leishmaniases with projects identified proactively for further exploration. The first success for DNDi will be the registration of the anti-malarial artesunate/amodiaquine co-formulation in early 2007 (see [1] for further information).

Workshop participants were asked to discuss all treatment modalities available and address the following questions and make recommendations:

• Based on available data, what should be the level of involvement of DNDi?

• Is there one treatment for all? Or do different forms of CL require different approaches?

• Is there a priority to address a particular form of CL?

The participants were urged to review, discuss (and disturb) present dogmas and consider current and long term opportunities to be captured in the report of the meeting. The output of the meeting including the recommendations to DNDi (short term and long term) will be used as the basis for a Strategy Document on CL to inform decisions for inclusion of projects in the DNDi Portfolio.

## Abstracts of presentations

Below are excerpts from the presentations of participants and the discussions that followed.

**Dr J. Alvar (WHO) **started the participants' presentations by reviewing the global public health importance of CL. A total of 11.8% of total worldwide DALYs by all leishmaniasis occurs in the Eastern Mediterranean countries, where CL is concentrated. In most cases, the per capita income in the leishmaniasis endemic-areas is substantially lower than the national per capita income.

He reviewed the relationship between poverty and CL in different countries where an estimated 90% of global cases occur (Table 2). In Kabul Afghanistan, there were 33,723 cases reported for the population of 6.3 million. The per capita income of highly affected areas was $160/year compared to the national per capita GDP of $190 for 2002. He stressed that disfigurement caused by CL leads to stigma, isolation and barrier to marriage, especially for girls. Patients are reservoir of infection and delay in treatment increases the chance for transmission. Proximity of cases with uninfected individuals creates a risk of transmission and crowding is one aspect of poverty in many urban communities. CL has creates a burden on national economy. Seventy seven % of men in Ecuador believe CL diminishes their ability to work. The cost of treatment is high and in most cases beyond the means of affected people. Dr Alvar urged DNDi to get involved in this truly neglected disease and particularly consider ACL in South-, and South Western countries of Asia.

**Table 2:** Relationship between poverty and CL incidence

**Poverty and other indicators for 90% of global CL cases**				
	% Population earning < 1$/day	Years of Life Expectency	% With access to improved water sources	Literacy rate (for >15 yrs old)

Afghanistan*	?	46	13	36
Algeria	<2	69.2	89	67.8
Bolivia**	14.4	63.2	83	86
Brazil	9.9	67.8	87	87.3
Colombia**	14.4	71.8	91	91.9
Iran	<2	69.8	92	71.1
Peru	15.5	69.4	80	90.2
Pakistan	13.4	60.4	90	44
Saudi Arabia	?	71.9	95	77.1

Sudan*	?	55.4	75	58.8

* = Categorized as Least Developed Country (LDC)

** = Not included in earlier lists of countries with highest

leishmaniasis burden, incidence has increased substantially

Only two countries are listed as LDC, however other countries have pockets of poverty ranging from <2% to 35% of the population. Inclusion of non-economic indicators provides a more comprehensive picture of poverty. (adopted from L. Alvar, WHO).

**Discussion: CL is mostly the disease of poor people. It is a spectral disease and represents many forms. Some forms are self healing but leave scars with strong social stigma and marginalization. Other forms are non-healing or chronic with sequels leading to disfigurement or mutilation of face with severe social and economic consequences. There is not adequate attempt to develop new treatments. DNDi is urged to get involved and develop affordable, efficacious and safe short treatments (preferably topical for uncomplicated CL) that do not require demanding health system infrastructure**.

**A. Llanos-Cuentas **described the CL situation in Peru, where disease distribution was compared and contrasted with certain social indicators in various areas of the country. He concluded that the primary factors which increase the risk of transmission and disease are ecology, and social factors such as the number of physicians/10,000 population, fertility rate, schooling, and poverty (income), while parasites, vectors and reservoirs are secondary factors. He indicated that the distribution of CL in Peru can almost be superimposed by areas of low social factors. Loss of income due to CL is an important factor in poverty.

**Dr A. Rabello **presented the situation in Brazil. CL is considered to be an important public health problem in some states as the number of cases has increased many folds in recent years. An interesting observation is that the incidence of disease is inversely related to GDP per capita for different states (Fig. 3). The cost of treatment with antimonial in Brazil is about US$ 150. However, Ambisome is used at extremely high cost. It was estimated that US$ 500,000 was spent to treat 95 patients; the cost to treat with antimonials for 35,000 patients was US$ 2.5 M. This is a considerable burden on health system in Brazil.

**Figure 3:**
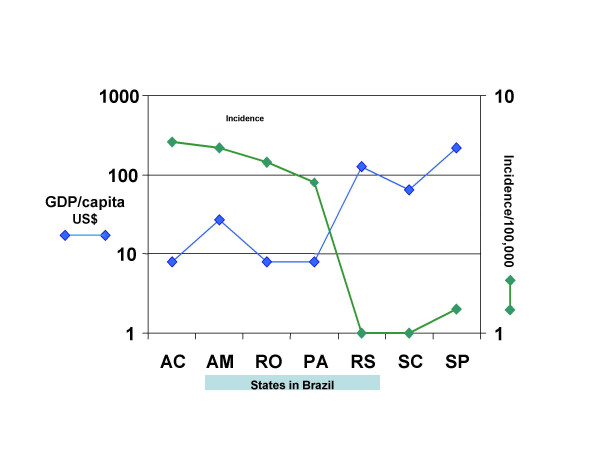
**Poverty and CL**. Inverse relationship of incidence of CL (green diamond) to GDP/capita (black diamonds) in various states of Brazil. AC = Acre, AM = Amazonas, PA = Para, RO = Rondonia, RS = Rio Grande do Sul, SC = Santa Catarina, SP = Sao Paulo. (Adopted from A. Rabello).

**Dr N. Rizzo **described the situation in Guatemala, where CL is rated fourth as the most frequent condition and sixth as most serious disease in the area. There is no official support for diagnosis, treatment or follow up. The cost of treatment by antimonial is about $252.00/patient which is the equivalent income of 2.4 months on average. CL is a rural disease and only 35% of rural population has a formal job with minimal salary of US $5.15 per day. Even when treatment is offered free of charge, the cost to patients is about US $82 or equivalent to 73% of an average monthly salary.

**Dr F. Meheus **had worked on VL in the Indian subcontinent and reported that the cost before starting treatment could be substantial. In India there is an average of 7 visits before starting treatment. In addition, the cost of treatment with antimonial is a great burden and beyond the means of most patients. For CL the cost of treatment is lower as there is no hospitalization, nevertheless it is not affordable for most patients.

**Discussion. The presentations on socio-economic studies were discussed together. There is a paucity of socio-economic studies, the available indicators used (DALY's and mortality) are not exactly applicable and do not reflect morbidity caused by CL. Well designed studies and relevant indicators are need**.

**Dr A. Magill **discussed the adverse events associated with current chemotherapy and stressed that safer and more efficacious drugs are urgently needed. Globally, the first line drug against CL and its various sequels is penta-valent antimonials (Sb^+5)^. A full course of Sb^+5 ^is poorly tolerated, requires careful monitoring to prevent severe and serious adverse events (SAE), is given parenterally which is inappropriate for uncomplicated CL. In the worst scenario, it can cause death if not carefully monitored, transmit blood born infection. Antimonials are contraindicated in pregnancy, patients with significant renal and hepatic disease and diabetes. Common adverse events are myalgia, joint stiffness, malaise, anorexia, transaminitis, increased amylase/lipase, and bradycardia with ECG changes of prolongation of QT interval and T-wave inversion. Less common AEs include hepatotoxicity, hemolytic anemia, nephrotoxicity, pancreatitis. Angioedema, anaphylaxis, post-Rx zoster have also been seen but only rarely.

Other medications that have been used as second line treatments or are in use without adequate assessment of their usefulness have not been superior to replace Sb^+5^. These include topical application of thermotherapy, cryotherapy, intralesional Sb^+5^, topical paromomycin ointments/gels and topical imiquimod together with other treatments. The systemic treatments include amphotericin B, pentamidine, oral azoles, oral topical miltefosine, dapsone, allopurinol, and many others. These are either not fully effective or affordable and are too toxic or require long-term administration. There have been numerous trials published however a new, non-invasive, efficacious and affordable treatment is urgently needed.

**Discussion: Clearly the present available medications are toxic and side effects are accepted because there are not many choices. There is a need to develop safe, efficacious non-invasive and affordable new treatments for different forms of leishmaniasis**.

**Dr P. Olliaro**'s critical review of published studies from 1994–2005 on clinical trials of CL was presented by F. Modabber (he was unable to attend). A total of 75 articles were reviewed of which 42 trials were controlled. Twenty seven trials without control (C) were published, which for a generally self-healing disease for many CL's provide very limited information.

Of the 42 controlled, 20 were randomised, double-blind with placebo control, 18 were either not randomised, else were not double-blind. These are also subject to bias outcomes. A total of 4650 subjects (mean 111/study, ranging from 20 – 400) were enrolled of whom 90% (4207) were evaluable in per-protocol analyses (Fig 4).

**Figure 4:**
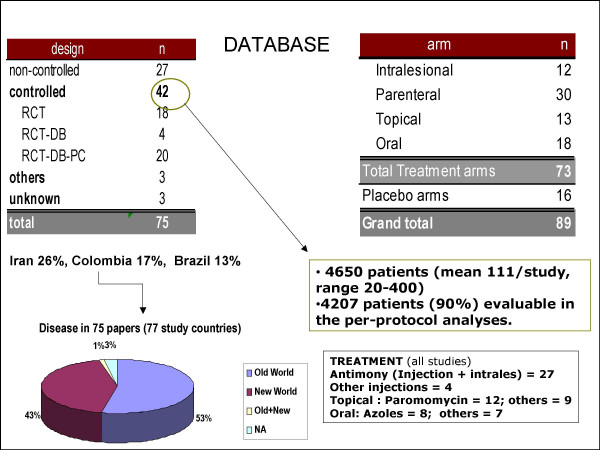
**Analyses of published clinical trials involving CL**. Of 75 studies only 20 were randomized, control trial (RCT), double blind (DB) with placebo control (PC). There were 27 trials that had no control arm. For a self healing disease, a control arm is necessary if it is ethically correct. There is a need to improve the quality of clinical trials.

In conclusion, the paucity of well designed trials is apparent. This has lead to our inability to judge the true efficacy of many agents and their comparable value. DNDi is urged to create a platform for establishing standard trial designs and enhancing capacity for trials with highest current Good Clinical Practices guidelines. In particular, Dr Olliaro's report emphasises that studies for CL should be comparative (or placebo controlled whenever possible), randomised and double-blind. As topical placebo may have an effect on the healing process, the natural history of the disease in the trial area must be established, else, if considered ethical, delayed rescue design should be considered, with CL forms that do not evolve rapidly or have severe sequels. The design should be amenable to individual patient data (IPD) for meta analyses. As a certain % of CL lesions heal spontaneously (even after years), the duration of the disease as an inclusion criterion is essential. Standardised definition of case and outcomes are very important if studies are to be compared. As well, reporting should be uniformed as per CONSORT statement (see ). It is suggested to use survival analysis (Kaplan-Meier) plots for rate of healing, use hazard rates, compare with a logrank test or the Mantel-Haenszel chi-square. Sample size should be adequate to provide sufficient power for multivariate analyses allowing influence of age, sex, type and number of lesions, previous duration of illness, etc. DNDi could prove a great impetus by developing a platform for CL clinical trial to increase the standard of evaluation of new treatments

**Discussion: Lack of adequate drive to develop new treatment for CL is apparent from the published data. Designs of studies are highly inadequate, variations in case definitions and end points make comparison of trials impossible. Contradictory results may well be due to these inadequacies. DNDi is urged to develop a platform for communication on clinical trials of CL globally and to develop standardized model protocol and increase capabilities for performing GCP-ICH level clinical trials**.

**Dr S. Meaume **described several approaches for promoting healing of cutaneous lesions, based on her knowledge of the process of wound healing. Initially, a blood cloth is formed, polymorphs migrate to wound margin, epithelial cells migrate over the wound surface and stimulated by macrophages lay collagen fibres and, when re-epithelialization is complete, the blood cloth is discharged. The first objective of therapeutic approach of wounds is to promote these processes. The classical paper of Winter GD [2] showed that a moist environment accelerates wound healing. The second objective is the closure of the wound and filling of the depression created in order to minimize scarring. Hydrocolloids with an internal layer of carboxymethyl cellulose and an external layer of polyurethane foam are used in all the stages of healing. When wound exudate is moderate, polyurethane films are adequate. Profuse exudation requires alginates, because of their very high absorbency. Hydrocellular dressings and foam have about the same use as alginates. Hydrogels contains more than 80% of water that can be liberated in the wound. Charcoal dressings reduce exsudates and bad smells. Dr Meaume considered that a polyurethane films widely available and affordable would be best for use on lesions of CL. Potential skin irritation induced by repeated application of adhesive dressings would be easily controlled by application of widely available affordable ointments (Mitosil) and/or local cortisone on the skin surrounding the CL lesion, when indicated.

**Discussion: To promote healing of CL lesions, methods used in wound healing should be employed. Affordable occlusions are available that can safeguard against super infection and create a moist environment for accelerating healing process and reducing scars**.

**Dr G. Milon **attracted the attention to the persistence of parasites in developmental stage following the control of cell-cycling *Leishmania *amastigotes in C56Bl/6 mice. Are these amastigotes non-cell-cycling *Leishmania *that persist in the upper dermis and are those the only ones that are transmissible to the sandfly vector? These parasites are not readily quantified by classical techniques, or their quantification is still expensive (quantitative Real Time PCR). Dr Milon warned that – as in this mouse model-, humans may harbour *Leishmania *that were reaching this developmental stage after patients were treated. If so, then it could be explain why long after recovery from the primary lesion some could enter the cell-cycle phase and create secondary lesions. In addition, these quiescent parasites could be transmissible to the sandflies. A treatment modality that could eliminate these "dormant/quiescent non cycling parasite population" might be needed for long term control of anthroponotic (or canine) leishmaniasis in endemic areas.

**Dr T. Lang **described a system (and later demonstrated it to some participants) by which the course of *Leishmania *infection can be monitored in the ear lobe of C56Bl/6 mice that received 10^4 ^luciferase – expressing *L. major *metacyclic promastigotes intradermally. The bio-reagent can then be measured quantitatively during the rise and fall of the parasite population (Fig 5). Ongoing explorations for detecting very low number of parasites are expected to allow the detection/quantification of the dormant population in the healed ear and in distant cutaneous sites. Dr Lang indicated that this system would be adaptable to screen treatment modalities, new compound, and drug combinations and drug+ immuno-modulator. The system would be able to monitor the initial fall of parasite burden but also very soon persistent organisms over a long period. He also noted that a quantitative real time PCR is now also available for addressing this latter issue.

**Figure 5:**
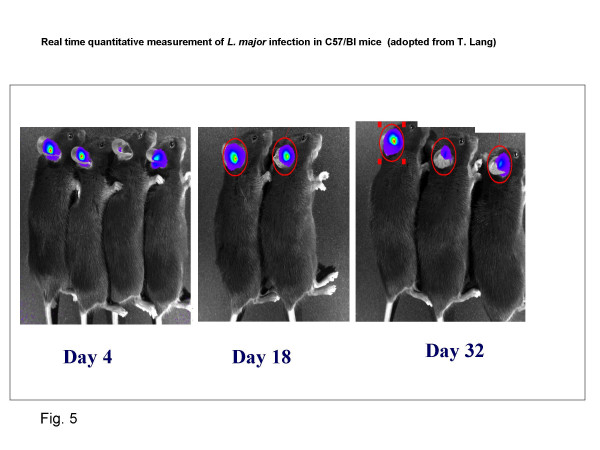
**Real time quantitative measurement of *L. major *infection in C57/Bl mice**. Mice were infected with 10^4 ^luciferase – expressing *L. major *metacyclic promastigotes intradermally. The bio-reagent can then be measured quantitatively during the rise and fall of the parasite population at various times. (adopted from T. Lang's presentation)

**Discussion: Measurement of parasite loads in experimental animals used for treatment evaluation has been labor-intensive. The new technology of using luciferase expressing *Leishmania *and bio-photonic imaging system provides a strong tool to study the efficacy of various treatments in the *L. major*/C57Bl/6 mice. There is a need to develop other animal models for different *Leishmania *species using this technology. Some immuno-modulators (cytosine-phosphate-guanosine oligodeoxynucleotides, CpG's) are species-specific hence it should be noted that although the general approach can be evaluated, the individual compounds would need to be identified for human use**.

**Dr S. Croft **reviewed compounds and drugs with some potential for treatment of CL. Based on experimental *in vivo *data there are disappointingly few candidates. These are inhibitors of sterol biosynthesis (anti fungal drugs), propyl-quinolines, Chalcones (licochalcones), naphtoquinines (buparvaquone, see Dr Garnier, below), diamidines, bisphosphonates, azithromycin, and saponisides. DNDi's VL strategy which includes synthesis and screening will generate new chemical entities. Those with activity against *L. donovani*, should be tested against CL causing organisms.

**Discussion: With limited resources available, a specific discovery program for CL producing *Leishmania *species does not seem justified at this time. However, lead compounds and drugs identified through the discovery strategy of DNDi for VL should be tested against CL-producing species**.

**Dr B. Arana **reviewed the clinical trials with oral drugs against CL caused by different *Leishmania *species. These included miltefosine, ketoconazole, itraconazole, fluconazole, allopurinol, dapson and oral zinc sulphate. Most trials were open, non-controlled, inadequately explained or reported with small sample size. As these were done in different clinical scenarios, comparison of results were not possible. Generally the duration of treatment is long (6–9 weeks) with oral drugs i.e, 6–9 weeks for ketoconazole, 4–9 weeks for miltefosine. The cost of azoles and miltefosine are prohibitory, allopurinol is not effective alone and was shown to be beneficial only in combination with Sb^+5^.

**Discussion: Azoles are still expensive and will probably remain so after their patent protection is over. The long duration of treatment required make their use less desirable. Alternative drugs should be sought. The development of miltefosine for different indications, including CL in different foci is ongoing outside DNDi. DNDi should only monitor these developments**.

**Dr Afif Ben Salah **presented the outline of a Phase 2 double-blind randomized, placebo-controlled (DBRPC) clinical trial of WR279396 (a new formulation of paromomycin) recently being conducted in Tunisia and France on 92 patients with CL caused by *L. major *(ZCL). He emphasized the problem of cost, lack of efficacy and poor availability of Sb^+5 ^in the region, Maghreb (Tunisia, Algeria, Morocco), with an estimated 300000 CL cases caused by *L. major *each year. Patients were treated for 3 weeks with the ointment (or placebo) and covered by an adhesive polyurethane dressing. Results are expected in early 2007. A second Phase 2 study is ongoing to evaluate the influence of occlusion and a Phase 3 pivotal trial is being planned. This new paromomycin formulation (i) is manufactured with active compounds that have been in human use for decades, (ii) has been developed according to industry/FDA standards, (iii) should be affordable, (iv) should be tested for efficacy against other CL clinical forms/infecting species, particularly *L. tropica*, should the ointment prove to be efficacious.

**Dr. Max Grögl **summarized the results of a 2-day meeting (15^th^–16^th ^of June) in Paris that brought together representatives from key organizations interested in the new product of Walter Reed Army Institute Of Research (WRAIR), the new ointment, WR279396. The meeting was organized by IP Paris, IP Tunis, and WRAIR and focused on a second generation topical treatment for CL. The format of the meeting was a combination of summary presentations of the development phases of WR279396 followed by an open dialogue among the meeting participants (DNDi, TEVA Pharmaceuticals-USA, Pasteur Institute, Tunis, MOH Tunis, Institut Pasteur Paris, US Army Medical Research and Materiel Command-USAMRMC) to arrive at goals and objectives for the product and the requires action plans necessary to achieve these goals. DNDi representatives (Drs Croft and Don) were invited and participated. Dr. Grogl indicated that for more than a decade, the USAMRMC has been actively engaged in the development of WR279396, a new topical therapeutic for the treatment of cutaneous leishmaniasis. That the steadfast support and combined work from the triad of organizations comprised of the Institut Pasteur Tunis, Institut Pasteur Paris, and US AMRMC, has offered them with clinical capabilityies that they hope will culminate in WR279396 becoming the next new approved drug to treat CL. Dr Grögl indicated that DNDi will be kept abreast of all developments, and that should the results of the Phase 3 trial mentioned by Dr Ben Salah, above, indicate sufficient efficacy, the ointment would be widely available for treatment of CL caused by *L. major *to all those who need them. The ointment would be initially registered in the U.S., however after registration it is planned to transfer the technology for production of the ointment to Tunisia.

**Discussion on the new WRAIR ointment: The results of phase 2 and the pivotal phase 3 trials are crucial and anxiously awaited. The close contact between WRAIR and DNDi on development of this ointment is highly encouraged and the offer of availability of the ointment at low cost following its registration as well as transfer of technology is much appreciated**.

**Dr A. Rabello **presented laboratory and some preliminary clinical results of a new gel formulation of paromomycin. The gel consists of 10% paromomycin in 1.5% hydroxiethylcellulose in water. Absorption of paromomycin in this gel to skin was 205X increased as compared to that of a paromomycin ointment in a modified Franz diffusion chamber, using mouse skin. Application of this gel along with systemic antimony 5 mg SbV/kg was as efficient as 20 mg Sb^+5^/kg in *L. amazonensis*-infected mice. The administration of the gel alone was as efficient as 20 mg Sb^+5^/kg in *L. braziliensis*-infected hamsters. The technology of preparation of this gel has been transferred to a pharmaceutical company in Brazil and pre-clinical development has been initiated and a clinical trial is being set-up (pending ANVISA, the Brazilian Regulatory Agency, approval) with the support of Fundação Oswaldo Cruz in Belo Horizonte and Três Braços – Bahia, to test this gel in humans.

Dr Rabello also reported that the results of a preliminary trial of oral azithromycin in patients with CL caused by *L. braziliensis *in an open cohort of 16 patients are very encouraging, confirming previous *in vitro *data.

**Discussion: The experimental results are very encouraging. Much discussion was focused on the ethical consideration of topical treatment of CL caused by *L. braziliensis*. The dogma that systemic treatment is required to prevent development of mucosal lesions was questioned. Dr P. Buffet presented analyses that indicated that first line topical treatment of these CL cases might be both ethical with respect to risk/benefit and cost/benefit consideration. In addition, it has been shown that topical heat treatment of lesions in Brazil, induces a systemic immune response and should not be considered as only a local reaction**.

**Topical treatment of lesions caused by *L. braziliensis *is probably both ethical and desirable. An expert review and analysis of peer reviewed literature would be helpful**.

**Dr A. Khamesipour **presented the results of 2 published trials conducted in Iran with the ointment containing 15% paromomycin, 10% urea in white paraffin, originally developed by Neal and colleagues. The first was a two arm, DBRPC trial of 2-week treatment (2 WK) on a total of 251 patients with lesions < 3 months of duration. It showed a transient but significant reduction in parasitological cure at the end of treatment, but no advantage in cure rate (complete re-epithelialization). The Second was a DBRPC trial of 4-week treatment (4-WK) vs. 2-WK active treatment plus two weeks placebo on 216 evaluable patients from a focus of ZCL, the same as the first trial. There was a significantly (p = 0.05) higher clinical cure rates for 4-WK group (80/108, 74%) on day 29, vs. 64/108 (59%) for 2 WK group. On day 45, there was a significant (p = 0.005) difference between the two groups with respect to parasitological and clinical cure (44%vs.24%) in favor of 4-WK. On day 105, the results favored those with 4-week treatment but the difference was no longer as clearly significant (p = 0.06). It was stressed that the duration of the disease prior to initiation of CL trials is crucial, as ZCL is by and large a self healing disease within 4–9 months. From this trial, the true efficacy of the ointment should be considered to be only about 35–40% (the difference between active treatment and placebo), as 30–40% would have self healed giving an efficacy of 75–80% total (Fig 6). This ointment may be considered as first line treatment for uncomplicated ZCL[4], which according to WHO should not be treated with Sb^+5^, a recommendation that is seldom applied as patients demand treatment. Preliminary trials of this ointment on ACL have shown no advantage in cure of lesions.

**Figure 6:**
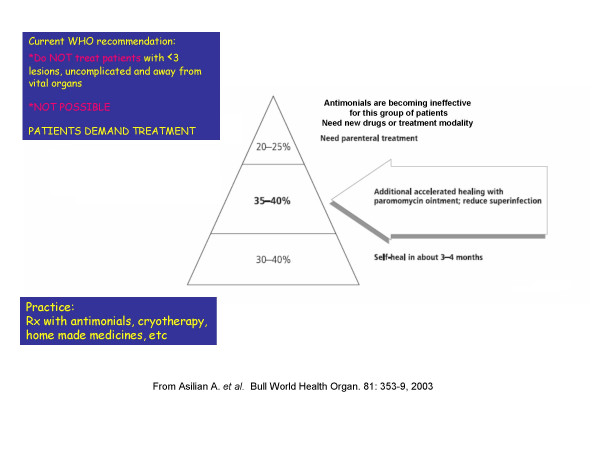
**The public health aspect of paromomycin-urea ointment**. The true benefit of the paromomycin (aminocidine) ointment in ZCL patients (caused by *L. major*) in Isfahan, Iran. About 30–40% self heal within 5–6 months from onset of disease, With an efficacy of the ointment about 35–40%, 75–80 would cure within this period leaving 20–25% of refractory patients who would need other treatment. This figure indicates the importance of a placebo (control) arm in all CL studies. In addition, the duration of lesion prior to enrolment into any trial is critical, since with time, more and more cases self heal.

**Discussion: There are indications that this ointment is not efficacious against ACL. However, one published trial **[3]**showed that topical paromomycin (P-ointment) twice daily for 15 days was significantly more efficient than oral ketoconazole in the *L. tropica *focus of Urfa, Turkey. At 4 weeks of treatment, 37.5% of patients were cured and 20% improved. No further follow-up data were provided. There are conflicting reports of efficacy of this ointment within Iran. Most trials suffer from inadequacies related to design, sample size and standardized definitions of case and endpoints. Only comparative parallel studies of different ointments can be used to evaluate different ointments. GMP preparation of all material used in phase 3 studies can not be over emphasized and results with home-made material should be disregarded**.

**Dr KW Stahl **presented the results of two approaches recently used in Afghanistan (i) Coagulation and evaporation of *Leishmania *infested tissue by electro-thermo-cauterisation, (ii) Moist wound treatment with and without pharmaceutical chlorite DAC N-055. The efficacy of both approaches is being assessed with the view of developing an affordable, efficacious, safe and doable treatment for the most unprivileged population of Afghanistan, living under very difficult conditions. In Afghanistan, there is at present no infrastructure to guarantee the quality control of drugs, their proper handling and use and the problem of ACL continues to impose heavy morbidity (see Alvar, above). Dr Stahl's group at the German Medical Centre, Kabul, have chosen a topical physico-biochemical approach to CL treatment rather than Sb^+5^, which would not require blood tests for monitoring side effects, or increase the chance of selecting resistant organisms already seen in the region. Local anaesthetics were used while applying electro-thermo-cauterisation as it can be painful. Occlusion with a polyurethane dressing with/without a gel was efficient in most patients. Parallel study of parasite loads using the limiting dilution technique showed that parasite loads at the beginning of treatment was sometimes very high (up to almost 10^9 ^parasites per gram). Most patients healed before D45 after a single application (Fig 7). As the study is still ongoing, the exact efficacy of this approach can not be given at this time, but the results are very encouraging. Over all, satisfactory results have been obtained under the difficult conditions with very limited resources. Should the results indicate, efforts should be made to bring pharmaceutical chlorite DAC N-055, a very inexpensive drug in Germany, to the international pharmacopoeia.

**Figure 7:**
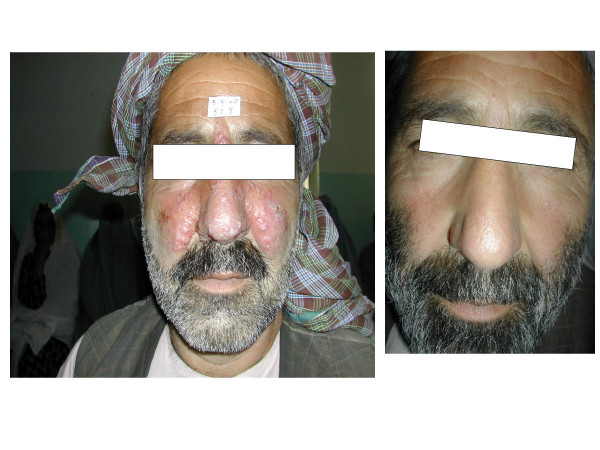
**A patient (Afghanistan) with lupoid CL before and after DAC N-055 therapy**. (Adopted from K-W Stahl, A F Jebran, F M Amin, R Steiner, A G Faramol, F Mahfuz, C Kurzmann, U Schleicher, C Bogdan – unpublished. Presented by K-W Stahl).

**Discussion: The efficacy of treatment with electro-thermo-cauterisation is not known yet, but will be known when the studies are complete within the next year. At this time the group did not seem to recommend use of equipment and repeated contact with a health worker. It is hoped that new innovative strategies can be developed for CL in remote areas as well which would make the use of electric equipment difficult if not impossible. However the use of DAC N-055 and moist occlusion was interesting. The work of German Group in Afghanistan is commendable. Afghanistan is the focus of one the worst ACL epidemics in history. Collaboration and communication of the Group with the proposed DNDi clinical trial standardization platform is highly recommended**.

**Dr T. Garnier **presented data on Buparvaquone (BPQ) topical treatment of *L. major *infection in Balb/c mice. BPQ was selected because of its *in vitro *and *in vivo *activities against several *Leishmania *species and its physical-chemical properties. Ideally, a topical drug candidate should have a small molecular weight (<500 Daltons), low melting point (m.p.), low hydrophilicity and have few functional groups capable of hydrogen bonding. BPQ has several physicochemical properties suitable for topical delivery. It has a MWT of 326.43, m.p. 182.2 – 183°C, 2 hydrogen bond accepting and 1 hydrogen bond donating groups, poor aqueous solubility and therefore suitable for topical formulation. Less lipophilic phosphate prodrugs were designed with the aim of altering physicochemical properties and improving BPQ skin permeation. Two BPQ prodrugs have been synthesized, namely buparvaquone-3-phosphate (BPQ-3-phosphate) and 3-phosphono-oxymethyl-buparvaquone (3-POM-BPQ), both of which exhibit a significantly increased aqueous solubility compared to BPQ, while not greatly increasing molecular weight. Various carriers ready for human use or in development were used for delivery of these compounds and tested in the *L. major*/Balb/c model. Both a BPQ hydrous gel and a BPQ water-in-oil emulsion significantly reduced both parasite burden (P < 0.05, 22 days post infection; as determined by real time PCR) and lesion size compared to the untreated control (P < 0.0001, 13 days post start of treatment) see Fig 8.

**Figure 8:**
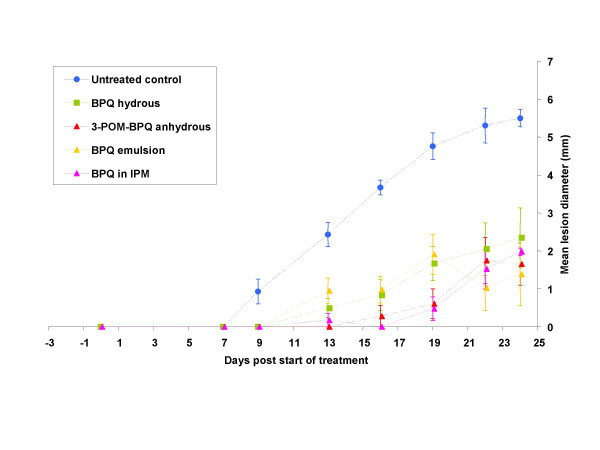
**Response to topical treatment with different buparvaquones (BPQ)**. BALB/c mice were infected with 2 × 10^6 ^*L. major J118 *promastigotes and 20 daily topical application began 3 days post-infection. Both a BPQ hydrous gel and a BPQ water-in-oil (w/o) emulsion significantly reduced both parasite burden (P < 0.05, 22 days post infection; as determined by real time PCR) and lesion size compared to the untreated control; P < 0.0001, 13 days post start of treatment, (adopted from T. Garnier).

**Discussion: BPQ is a registered drug in veterinary use for treatment of *Theileria *infection. Hence much information of its safety profile exists. It would be useful to follow development of a topical preparation of this drug for CL as it is active against many CL producing species. It has not been tested against *L. tropica *and it would be of interest to see if it is active against this species**.

**Dr Matlashewski **introduced Imiquimod, (1-[2-methylprophyl]-1H-imadazo [4,5c] quinoline-4-amine), which was developed by 3 M company for the treatment of cervical warts due to human papilloma virus. It is marketed as Aldara (5% imiquimod cream). Imiquimod and its more active analog, R848, are synthetic low molecular imidazoquinoline compounds which are potent immuno-modulators through activating Toll-Like Receptor7 (TLR) and TLR8 on human antigen presenting cells including macrophages and dendritic cells (Fig 9). Activation of TLR 7/8 with R848 results in secretion of pro-inflammatory cytokines including IL-12 which leads to the polarization of naïve T cells into a Th1-type cell. This leads to polarization of the response toward Th1 response, needed to kill intracellular *Leishmania*. Imiquimod can activate macrophages to kill intra cellular *Leishmania*. Following the initial successful results against *Leishmania *in laboratory, it was quickly possible to proceed to clinical trials since imiquimod was already used in humans.

**Figure 9:**
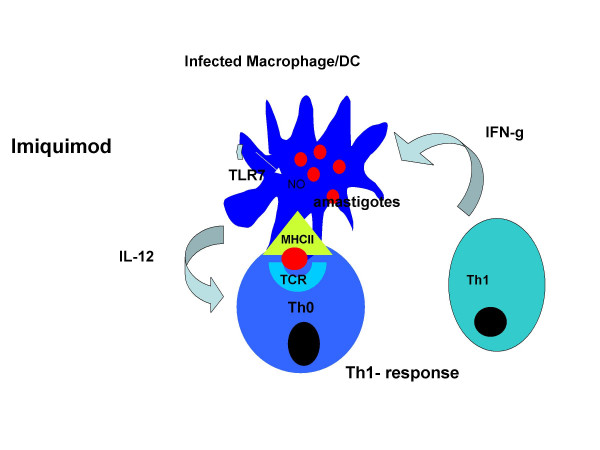
**Schematic representation of the mode of action of Imiquimod**. IFN-γ = Interferon-gamma, IL-12 = Interleukin-12, MHC-II = Type 2 major histocompatibility antigen, NO = Nitrous oxide, TCR = T-cell receptor, Th1 = T-helper cell type 1, Th0 = Undifferentiated T-cells, TLR-7 = Toll like receptor-7. (presented by G. Matlashewski)

Two clinical trials have been concluded in Peru involving the treatment of cutaneous leishmaniasis with topical Aldara (5% imiquimod, donated by 3 M pharmaceuticals) [5,6]. These studies demonstrated that combining imiquimod treatment with the standard pentavalent antimony treatment is safe and significantly increased the cure rate compared to treatment with antimony alone in patients which have failed a previous treatment with antimony (non-healing patients). – see Llanos-Cuentas, below. Additional clinical trials in cutaneous leishmaniasis patients which have never before been treated is now ongoing with DNDi support. This involves (i) combining topical imiquimod (Aldara) and iv injections of Sb^+5^; and (ii) combining topical imiquimod (Aldara) with topical paromomycin. The second trial involving paromomycin is potentially important because it is designed to develop a combination topical therapy which would replace the need for iv injection of antimonials. Considering imiquimod mode of action, it seems that it can act as an adjuvant for therapeutic as well as prophylactic vaccination. Further studies are planned to test this possibilities.

**Discussion: Killing of the bulk of parasite by chemotherapy followed by stimulating the immune response in the right direction is the novel approach that DNDi should consider seriously. Imiquimod, having gone through safety and initial efficacy trials, is an obvious choice to be fully studies for maximum effectiveness. The patent protection of imiquimod will end soon and hopefully it will be produced as generic and become affordable. It also be of interest to test the adjuvant potential of imiquimod should it be necessary to use leishmanial antigens for immuno-chemotherapy or immunotherapy**.

**Dr A. Llanos-Cuentas **described the trials of meglumine antimoniate ± topical imiquimod for cutaneous leishmaniasis in Peru. [5,6] In a randomized double blind clinical trial 40 subjects with clinical resistance to antimony were recruited. All subjects received meglumine antimoniate (20 mg/kg/day im or iv) and were randomized to receive either topical imiquimod 5% cream (Aldara; 3 M Pharmaceuticals) or vehicle control every other day for 20 days. Lesions and adverse events were evaluated during treatment and at 1, 2, 3, 6, and 12 months after the treatment period. All but 2 subjects completed therapy. Mild adverse events were reported by 73% of the subjects, but only erythema occurred more commonly in the imiquimod group (P < 0.02). Lesions resolved more rapidly in the imiquimod group: 50% of the imiquimod group achieved cure at 1 month after the treatment period versus 15% of the vehicle cream group (P < 0.02); 61% of the imiquimod group at 2 months versus 25% of the vehicle cream group (P < 0.03); and 72% of the imiquimod group at 3 months versus 35% of the vehicle cream group (P < 0.02). Residual scarring in the imiquimod group was less prominent than in the vehicle cream group (Fig 10). In conclusions, combined antimony plus imiquimod treatment was well tolerated, accelerated healing of lesions and and improved scar quality. This therapy may have particular advantages for subjects with facial lesions.

**Figure 10:**
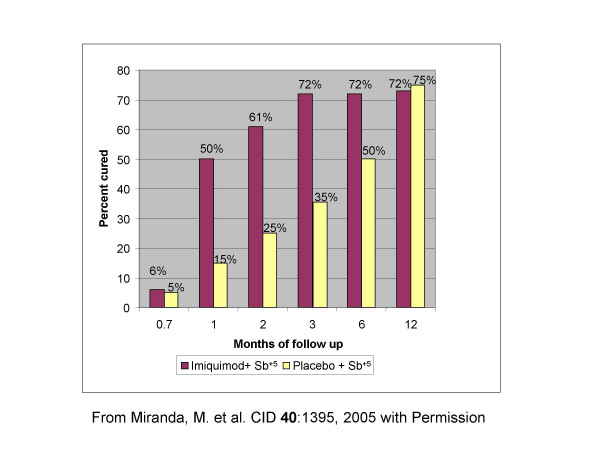
**Response to Immunochemotherapy of CL in Peru**. Double-blind randomized placebo-controlled trial of topical Imiquimod plus antimony vs placebo plus antimony. Forty subjects with clinical resistance to antimony received Sb^+5 ^(20 mg/kg/day im or iv) and were randomized to receive either topical imiquimod 5% cream (Aldara; 3 M Pharmaceuticals) or vehicle control every other day for 20 days. With permission from Miranda et al. Clin. Infect. Dis. 2005, 40: 1395.

In addition to the original open study on 12 patients in Peru which produced very encouraging results, another study conducted in Iran was also mentioned which showed some beneficial effect (Meymandi, personal communication). However there is one report from Iran on CL caused by *L. tropica *which showed no added effect by imiquimod to antimonial therapy (see Khamesipour, below).

**Discussion: Based on previous positive responses, additional studies with different doses would be of interest. In addition if imiquimod is active as an adjuvant, its use in therapeutic vaccines should be considered**.

**Dr A. Khamesipour **reported on the results of two trials in Iran. First was a three arm trial involving imiquimod alone (27 patients), imiquimod plus intra-lesional antimonial (35 paitents) and intra-lesional antimonial alone (35 patients) for a period of 6 weeks. The previous history of treatment in the patients was not known and the study was not according to a clinical trial protocol, but collection of records of treatment history. The results indicated a decrease in size of erythema, induration and ulcer (end point). There was a slight but definite beneficial effect by adding imiquimod to antimonial (37% Imiquimod + Sb "responders" vs. 23% antimonial alone). The second trial was a randomised, double blind, two arms controlled trial of 4 week treatment of imiquimod + 2 week antimonial vs. 4 week placebo + 2 week antimonial in 119 patients with CL. The results were compared 4, 8 and 20 weeks after the initiation of treatment. There was no detectable difference between the two groups at any time point. It was concluded that with the regimen used, 4 weeks of treatment with topical imiquimod does not enhance the response to antimonials.

**Discussion: The outcomes of the two Iranian trials were different. This may be due to differences in the design, duration of treatment, history of patients, sample size, endpoint definition, causative organisms, etc. The diversity of responses seen here and is observed throughout CL trials around the world, further indicates the urgent need for standardizing clinical trials of CL**.

**Dr F. Modabber **reviewed clinical studies on therapeutic vaccines in cutaneous leishmaniasis. Convit and colleagues in Venezuela initiated immunotherapy of CL with killed *Leishmania *mixed with BCG. In his review of 11532 cases treated, Convit reported that cure rates varied between 91.2% – 98.7%; time to cure was similar or only slightly longer than chemotherapy with 3–4 injections but the cost and side effects were considerably reduced as compared to Sb^+5 ^[7]. Side effects of the therapeutic vaccine were confined to the site of reaction due to BCG. This approach has been used in Venezuela for years, while attempts are being made to improve the vaccine, since this is a crude preparation (autoclaved parasites) and BCG injections (used as a T-cell adjuvant) produce lesions and leave scars. This preparation however, has not been efficacious as a prophylactic vaccine against CL. Similarly, Mayrink and his colleagues have used killed *Leishmania *without any adjuvant in Brazil and Colombia, again showing no prophylactic efficacy. However, the killed *L. amazonensis *vaccine was highly efficacious when used as an adjunct to low dose chemotherapy. In a double blind, sequential enrolment of 100 patients (96 evaluable), two-arm trial, the efficacy of 8 mg/Kg/d Sb^+5 ^(low dose) for 4 cycles (10 daily injection followed by 10 day rest = one cycle) was compared to that of the low dose Sb^+5 ^plus 4 cycles of killed *Leishmania *vaccine. At the end of the treatment47/47 (100%) of patients in combined immuno-chemotherapy group were completely cured vs. 4/49 (8%) in the low chemotherapy alone group (Fig 11). With this impressive data and previous smaller trials, this preparation was registered in Brazil as a therapeutic vaccine, adjunct to low dose Sb^+5 ^treatments [8]. The advantage of this approach is the reduction in cost and side effects due to full course of antimonials, but the disadvantages are long duration of treatment and the requirement for daily injections.

**Figure 11:**
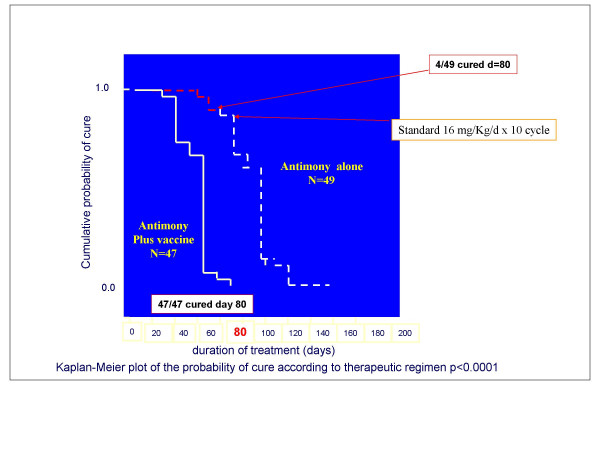
**Immuno-chemotherapy of CL caused by *L. braziliensis *in Brazil**. Double-blind controlled trial of Mayrink's vaccine (killed *L. amazonensis*) plus low dose (8 mg/Kg/d) Sb^+5 ^vs. low dose Sb^+5^. Patients received daily injections of the combination or drug alone for 10 days followed by 10-day rest (one cycle). All patients (47/47) were cured in the combination group after 4 cycles (80 days) vs. 4/47 in the chemotherapy alone group. The non-responders were rescued with full dose antimony. (Adopted from Machado-Pinto et al. *Int. J. Dermat*. 2002; **41**:73-8.)

**Discussion: All these results with the first generation vaccines (killed parasites) should be taken as proof of principle. It is well known in leishmaniasis that the immune response is an intricate part of recovery from *Leishmania *infection. It is therefore logical that with the modulation of the immune mechanism toward the protective immune response during therapy cure would be enhanced. The first generation vaccines are very inexpensive and safe as they have been given to tens of thousands of healthy humans in prophylactic trials as well as patients for therapy. However, they are crude preparations and not as yet fully standardized. In addition, the parasites are grown in media containing fetal calf serum, although it is possible to grow *Leishmania *in defined media. The alternative, the only one second generation vaccine, is in early clinical development **[9,10]**and not considering the cost which would most likely be prohibitive, the efficacy results would not be available for several years. Therefore, until a better vaccine becomes available, two avenues could be considered**

a- To a short course of chemotherapy add immuno-modulators without leishmanial antigens (i.e. Imiquimod, see Matlashewsky) or,

**b- To a short course of chemotherapy add a vaccine composed of an adjuvant and a standardized first generation vaccine produced with all current GMP requirements. Killed *Leishmania *plus CpG has been shown to be a potent vaccine in mice (see Sacks, below)**.

**It was considered that immunotherapy combined with short course of chemotherapy is an innovative approach and an opportunity not fully explored for leishmaniasis, though well advanced in oncology and other human diseases. DNDi is in a good position to launch this innovative modality for treatment of leishmaniasis**.

**Dr E. Khalil **was unable to attend. The recent trial of his group on immuno-chemotherapy of persistent PKDL cases in Sudan using alum-autoclaved *L. major *+ BCG (alum-ALM vaccine) was briefly presented by Dr Modabber. About 50% of treated VL patients develop PKDL and although 85% of the cases who are generally leishmanin skin test (LST) positive self heal within 1 year or respond to 30–40 days of Sb+5 treatment. Treatment of the remaining 15% in whom the disease persists for many years, is difficult, prolonged, and expensive and it often fails. The alum-ALM vaccine has been shown to be safe and induce primarily a Th1 response and LST conversion in almost 100% of Sudanese volunteers. Following the concept used in Venezuela and Brazil, the Alum-ALM vaccine was added to the standard Sb^+5 ^treatment for persistent PKDL patients. Following several small safety/immunogenicity trials of Alum-ALM alone or alum-ALM vaccine with or without antimonial in 5–8 persistent PKDL patients, a hospital based, randomized and double blind study was conducted with thirty patients, to further study the safety of vaccine and see if addition of the vaccine to the standard chemotherapy would enhance cure. On day 60, a total of 12/15(87%) patients in immuno-chemotherapy group (4 weekly injection of vaccine + daily Sb^+5^) were cured and the remaining showed considerable improvement. On the other hand only 8/15 (53%) of the patients who received antimonial alone healed completely within 60 days and the remaining failed to show any improvement (Fig 12). There were no relapse cases in the first group but 2 relapse cases in the antimonial group. The leishmanin skin induration increased significantly at day 60 in antimony/vaccine arm (*p *= 0.004) but not in the antimony-alone arm (*p *= 0.06). The *leishmania*-specific IFN-γ production *in vitro *on day 21 was significantly higher in the immuno-chmotherapy Vaccine arm compared to the chemotherapy alone arm (*p *= 0.002). The levels dropped to baseline by day 60.

**Figure 12:**
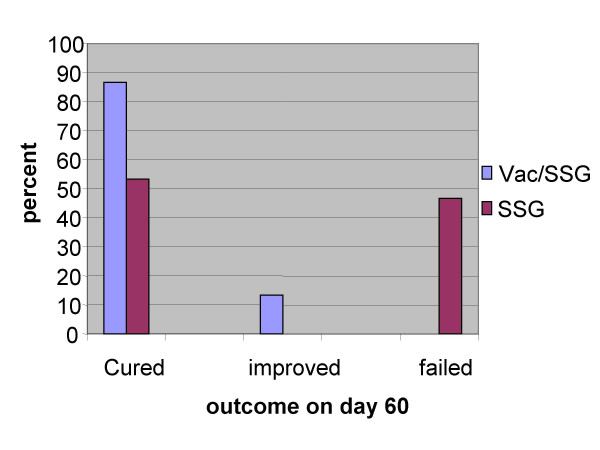
**Immuno-chemotherapy of persistent PKDL patients in Sudan**. Double-blind randomized antimony alone controlled vs. immunochemotherapy trial of 30 persistent PKDL patients. All patients received the standard daily injections of Sodium Stibo-Gluconate (SSB) for 40 days. Patients were randomized to receive 4 weekly injections of alum-precipitated autoclaved *L. major *(alum-ALM) + one tenth of normal dose of BCG or a placebo (BCG diluent). On day 60, 87% in the combined immuno-chemotherapy group were cured and the rest improved vs. 53% in chemotherapy alone group cured and the rest failed (efficacy = 72%, 95% CI: 0.8–1.18). By day 90 all patients in immunochemotherapy had cured vs. Adopted from Musa, A. & Khalil, EAG, et al.)

**Discussion: The results are highly encouraging, but this is a preliminary hospital based study and a larger trial is needed to see the exact efficacy and applicability of this approach in the field. PKDL cases are most likely the reservoir of infection and their treatment must be considered an important target for the control of VL in Sudan**.

**Dr D. Sacks **introduced Oligodeoxynucleotides (ODN) containing CpG motifs which have been used as adjuvants in experimental animals for prevention and treatment of CL[9]. The CpG's are rapidly internalized by immune cells (B cells, macrophages, dendritic cells and monocytes) and localize to endocytic vesicles where they interact with Toll-like receptor 9 (TLR9). TLR9-bearing cells recognize and respond to non-methylated CpG motifs (present at high frequency in bacterial but not vertebrate DNA), triggering an immune cascade characterized by polyclonal-B-cell activation, improved antigen uptake/presentation by antigen-presenting cells, and the secretion of chemokines and pro-inflammatory cytokines, including IL-12, IL-18, TNF-α, IFN-α, -β and -γ, that foster a strong Th1 response. In this regard, CpG ODN show promise as immuno-protective agents and as vaccine adjuvants. Extensive work in murine models of CL has shown that CpG ODNs can be used as an effective adjuvant with killed parasite and sub-unit vaccines against *L. major*, and as immunotherapy to reduce the severity and promote the clearance of *L. major *(Fig 13). Application of CpG soon before or after a live inoculum, can significantly reduce the pathology of the inoculum without reducing its protective potential against new challenge. It may therefore be considered as an adjunct to live parasite vaccine.

**Figure 13:**
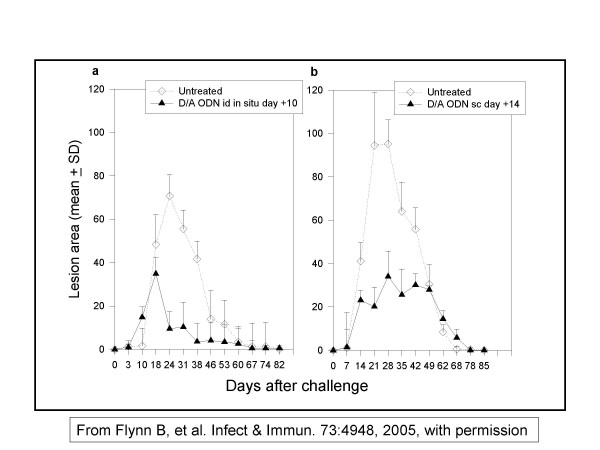
**Immunotherapy of CL lesions in macaque monkeys**. Systemic administration of oligo-deoxy-nucleotide (ODN) class D/A limits the size of lesions following an intradermal infection with *L. major*. Importantly, the reduced morbidity was not associated with a reduction in long-term immunity, as such treated macaques were still protected following a secondary challenge. This suggests a potential role for CpG ODN in *L. major *treatment. These findings support the development of clinical studies to assess the use of CpG ODN types D/A as immunoprotective and therapeutic agents. *(From Flyn B. et al Infect.& Immun. 73:4948, 2005 with permission*)

In addition, CpG administration has been shown to promote healing of an active lesion in experimental CL.

**Discussion: Development of CpG's as adjunct to chemotherapy of CL either alone or in combination with leishmanial antigens should be considered. The long lasting effect of CpG's when used with killed parasite vaccine in animal models makes them a good candidate in line with the view of reducing the need for patient – health worker contact discussed and included in the recommended TPP for CL**.

**Dr. Asrat Hailu **presented clinical characteristics of localized, mucocutaneous and diffuse cutaneous leishmaniasis in Ethiopia. About 85% of CL in Ethiopia is caused by *L. aethiopica*. The rest is caused by *L. tropica *and *L*. major. CL caused by *L. aethiopica *is generally self healing, however the sequels include diffuse and mucosal leishmaniasis in and a small % of cases. Lesions caused by *L. aethiopica *do not respond to Sb^+5^. The *in vitro *ED_50 _values for Sb^+5^, aminosidine sulphate and pentamidine were 25.3 μg Sb^v^/ml, 6.4 μM and 0.6 μM respectively. This *in vitro *study, demonstrated the poor activity of antimoniate on *L. aethiopica lesions*. Cryotherapy was apparently associated with healing but with a high incidence of intensely hypochromic scars. An efficacious, safe and affordable treatment is needed for CL in Ethiopia which is a public health problem due to stigma associated with severe and disfiguring lesions, long duration of illness, and severe sequels.

**Discussion: *L. aethiopica *is a unique organism unlike other species. Although it is a neglected disease, a special strategy may not be justified at this time. However, the new strategies for ACL should be tested against *L. aethiopica *lesion**.

## Conclusion and recommendations

The major conclusions and recommendations can be summarized as follows:

The global burden of cutaneous leishmaniasis was discussed with respect to

• Financial impact on the lives of CL cases, who in general are amongst the most under privileged population in endemic countries – the close association between poverty and CL incidence in many countries was noted

• The stigma associated with the lesion and scar of CL, particularly in young women leading to suffering and marginalization

• The financial burden and its impact on health systems of disease endemic countries

• Absence of a vaccine and lack of satisfactory (safe, affordable and efficacious) drugs

• The diversity of epidemiological characteristics of foci of CL in Africa, Asia, Europe and Latin America due to varieties in causative *Leishmania *species, their vectors and mammalian reservoir hosts

• Difficulties in control of CL due to lack of effective, affordable, easily implemented tools

• Lack of political will and resources to maintain control activities

• Lack of involvement of the pharmaceutical/biotechnology sector in the development of new drugs and vaccines

It was generally agreed that our knowledge of the incidence, prevalence and socio-economic impact of CL was limited. Prevalence is high, stigma associated with the disease is well known and the burden on individuals and governments are well recognized, but we have limited quantitative data. The disease is not generally reported and there is a paucity of socio-economic studies as well as high quality clinical trials in accordance with international standards of Good Clinical Practices (GCP). The first line drugs (antimonials, developed over 60 years ago) have serious side effects, require repeated injections, are expensive, not always available and in many parts of the endemic regions have become ineffective. Therefore, patients in endemic areas resort to whatever local treatments available, including burning, application of acids, herbal concoctions, curettage, etc. Victims are mostly children in endemic foci and lesions are frequently on the face.

Since CL comprises different disease forms that cannot necessarily be addressed by one and the same treatment, it was decided to set criteria through which CL with different (epidemiological) characteristics could be prioritized as targets for the development of a new treatment. These criteria included:

• Severity of the disease, including duration of disease, average size of lesions and number of lesions per patient and severity of scars

• Lack of response to available drugs

• The overall incidence and prevalence of the disease (based on available data)

• Sequelae of the disease, including leishmaniasis recidivans (a long-lasting relapsing form of CL often located on the face) and mucosal disease

• Public health aspects, including the impact of treatment of individuals on control of transmission of *Leishmania *spp, the etiological agent (s) of leishmaniasis

• Lack of major efforts in drug development

While it was unanimously agreed that new and improved treatments are needed for all forms of CL, some forms may represent a higher priority or opportunity for targeted R&D efforts.

Based on the above considerations, the anthroponotic CL (ACL) and its sequelae, Leishmaniasis Recidivans (LR) caused by *L. tropica *in the Old World was proposed as the priority target. In ACL, the cutaneous lesion may self heal in a year or longer but leaves an unsightly scar for the life of the patients. In 2–5% of cases, ACL becomes chronic or can develop into LR, a persistent presence of parasites that are able to expand and drive a cutaneous lesion around the original lesions during the course of the primary lesions or long after healing. Recent data indicate that ACL does not respond well to antimonials nor to some of the second generation drugs tested. It is highly prevalent in South-, South Western countries of Asia where it represents a major public health problem. The most affected populations live in Afghanistan, Pakistan, Iran, Iraq, Turkey and Syria. ACL is an anthroponotic CL, transmitted from human to human by the bites of sandfly vectors. Although in rare cases foxes and stray dogs have been shown to be infected by *L. tropica*, they are not considered as primary reservoir. Hence, based on epidemiological characteristics (sandfly specificity, urban-, chronic and the anthroponotic nature of ACL), treatment of cases is believed to reduce the size of parasite reservoir and thereby reduce transmission. The dynamics of the transmission need to be further elucidated.

As a second priority target, CL (and ML) caused by *L. braziliensis *in the New World was put forward. This CL occurs in most endemic countries of Latin America and is considered a major public health problem in Brazil, Bolivia, Peru, Venezuela and Guatemala. A dreaded sequel of this CL is mucosal/mucocutaneaous leishmaniasis (ML) that, unless diagnosed and treated early, will cause destruction of nasal septum, oral cavity, pharynges and larynges disfiguration and in some cases death. Many suicides or attempts have been recorded due to the stigma associated with ML. Prolonged and high dose treatment with antimonials is required for cure which is both beyond the means of most patients and associated with side effects due to the drugs. The best way to prevent ML is prevention or timely and adequate treatment of CL in the *L. braziliensis *endemic region.

To guide the R&D efforts, a Target Product Profile (TPP) was established for ACL (See Appendix 1. ACL-TPP.xls) with a strong emphasis on safety, efficacy, cost, quality of scar and reduced interaction with "health system". The new treatment should aim for a short treatment period and minimize the need for patient-health-worker contact.

Although a new treatment for ACL might not be effective against CL caused by other *spp *of *Leishmania*, some CL's may still respond to it. Similarly, a treatment being developed for CL caused by *L. major *may turn out also to be effective against ACL (e.g. topical aminoglycosides). So it will be critical to systematically assess possible new treatments for one form of CL in other manifestations of the disease.

Other than paromomycin and limited studies with miltefosine, there are no highly efficacious drugs or candidates in advanced stages of development, nor a screening program in place for the identification and evaluation of new chemical entities active against the pathogens that cause target CL. Moreover, there are no validated animal models for compound evaluation against *L. tropica*. The recently developed luciferase expressing *L. major *– C57Bl/6 mouse model allowing real-time measurement of parasite load simultaneously with monitoring lesion development and healing, provides a convenient way to assess new compounds and drug candidates, and would be very useful if adapted to various target spp of both Old World and New World *Leishmania*.

One promising approach with long term potential is to combine chemotherapy with immuno-modulating agents. The underlying mechanism of the body for controlling *Leishmania *multiplication and spread, and to achieve a stable self-healing process of lesions is an adequate immune response. It is the development of effector cells capable of eliminating parasites (predominantly initiated by CD4 and CD8 T lymphocytes that are tightly regulated by counter-inflammatory immune effector cells) that leads to cure and maintains the persisting parasites under control. Treatment of immuno-compromised patients is invariably associated with relapse. Therefore, an initial elimination of the *Leishmania *parasites by chemotherapy and the induction of a adequately balanced cellular immune response could lead to quick recovery and control of persisting non-dividing parasites. Immunotherapy can take the form of immunomodulators alone or in combination with *Leishmania *antigens.

There is currently no adequately effective vaccine; however, killed parasites when given together with some adjuvants (First Generation Vaccine, FGV). Although effective as prophylactic vaccine in experimental models, FGV do not protect against human CL. Nevertheless, FGV's have shown positive results as" therapeutic" vaccines for CL (Brazil & Venezuela), and for persistent PKDL patients in Sudan). These results are encouraging proofs of principle, but such crude preparations of killed parasite are not acceptable as final vaccines. More recently, defined antigens of *Leishmania *used together with Granulocyte-Macrophage Colony Stimulating Factor as adjuvant have been shown to cure drug refractory ML patients in a preliminary trial [10] One second generation vaccine (SGV, 3 recombinant *Leishmania *antigens + a new adjuvant) is in clinical development (phase I) as a therapeutic vaccine [11]. The early results of a safety and immunogenicity trial in Peru indicate that addition of Leish-111f +MPL-SE to chemotherapy ofML patients is safe and there is anindication thatthe time to cure may be reduced, compared to chemotherapy alone. Further studies are needed.

On the level of immuno-chemotherapy, in addition to cytokines, several chemical immunomodulators have been tested in combination with standard chemotherapy in humans (imiquimod) or various CpG's in mice and monkey models of CL. Currently, a clinical trial with imiquimod as adjunct therapy to antimonials is being carried out in Peru with support from DNDi. There are several CpG's in clinical trials for other diseases, i.e. cancer (CpG 7909 and Vaximmune of Coley; dSLIM of Mologen, etc.). Actilon is now marketed for hepatitis C treatment. The CpG's are species specific (except dSLIM, which is not and works in mice and humans) and different motifs act as agonists of various Toll Like Receptors (TLR). Due to our knowledge of immunology of leishmaniasis (at least in mice), the specific CpG-ODN's for targeted TLR can be selected to induce the desired immune response. The development of these agents should be followed closely and those appropriate for leishmaniasis should be pursued for for development against CL.

### Specific recommendations

CL in all its forms can be considered as a neglected disease, and organizations such as DNDi, WHO and TDR should place it on their list of target diseases for specific R&D efforts to develop improved control measures.

Efforts should initially focus on Anthroponotic CL due to *L. tropica *(ACL) and CL caused by *L. braziliensis*.

#### Target short term objectives

• Part of the reason for CL's neglect is related to the lack of reliable and meaningful data on the impact of the disease. Quality multi-center impact studies are needed to better assess the socio-economic burden and human suffering caused by the different forms of CL, with priority on ACL due to *L. tropica *and CL caused by *L. braziliensis*.

• The quality of clinical trials should be enhanced. It is essential to establish protocol templates in line with current Good Clinical Practices (c-GCP) and provide training and coordination. In addition, to increase the comparability of clinical trial outcomes, a standardized clinical trial methodology for CL should be developed, including guidelines on inclusion/exclusion criteria, clinical endpoints, test of cure, etc.

• Short course of chemotherapy together with immune modulators alone or immune-modulators plus *Leishmania *antigens (therapeutic vaccines) should be given serious consideration.

∘ The possible value of further investing FGV should be assessed, while the further development of SGV should be closely monitored, and supported if needed.

∘ The new CpG oligodeoxynucleotide adjuvants have encouraging results in experimental leishmaniasis. As soon as the safety profile is established in clinical oncology or other diseases, CpGs (with or without leishmanial antigens but with anti-leishmanial drugs) should be tested in clinical trials in CL.

∘ The field of immuno-modulators is a vibrant field of research, driven by their promise for the treatment of other diseases such as cancer. These developments should be closely monitored as new immunomodulators reaching the market could also show promise in CL.

• Clinical trials with drug combination should be initiated with the view of shortening treatment period and reducing cost and need for follow up.

• Based on the outcome of the clinical trials of the new topical amino-glycoside formulations, their efficacy against other forms of CL, in particular ACL should be determined.

#### Target long term objectives

• New chemical entities and drugs emerging from ongoing efforts to develop drugs for visceral leishmaniasis (VL) should be screened for activity against *L. tropica *and *L. braziliensis*. In case of good activity, the possibility and requirements for a parallel development for VL and CL should be explored.

• The new luciferase expressing *L. major *– C57Bl/6 mouse model should be fully exploited for assessing *in vivo *anti-leishmanial activities of drugs/compounds; even though it is not at present adapted to *L. tropica*/*L. braziliensis*. Attempts could be made to adapt this model for *L. tropica*. Other possible models (i.e. hamster and *L. braziliensis*) to be considered for development, but relevance to be decided on a case by case basis.

• The development of new vaccines and adjuvants/immuno-modulators with promise for CL should be closely monitored, with the view of potential use for immunotherapy of ACL (and later CL due to *L. braziliensis*). This would require further clinical trials.

• Topical treatment of CL is preferable to systemic interventions. Further research and studies to improve delivery and efficacy of topical treatment based upon new technologies needs to be supported.

Based on these objectives, two decision matrices were developed for short- and long-term objectives respectively (Figs. 1 &2).

## Conclusion

Cutaneous leishmaniasis is highly neglected, requiring new and improved treatment options that are effective against the different forms of the disease. A realistic assessment of the impact of control and treatment on public health and needs of patients is required. An improved and more standardized design of clinical trials should be developed, and efforts are needed to improve the quality of the clinical trials according to current GCP. New drugs and immuno-therapeutics are required to improve current treatment options; drug combinations involving either or both chemotherapeutics and immunomodulators have the potential to increase efficacy, reduce treatment duration and improve ease of use and compliance. There is a need for a coordinated response to address these issues.

## Abreviations

ACL, Anthroponotic cutaneous leishmaniasis

ALM Autoclaved *Leishmania major*

ANVISA The Brazilian regulatory agency

BCG *Bacillus Calmette-Guérin*

BPQ Buparvaquone, 3-POM-BPQ = 3phosphono-oxymethyl-buparvaquone

CL Cutaneous leishmaniasis

CpG, Cytosine phosphate guanosine oligodeoxynucleotide

DALY Disability adjusted life year

DBRPC Double blind, randomized, placebo-controlled

DNDi Drugs for neglected diseases initiative

FDA The United States food and drug administration

FGV First generation vaccine (whole parasite)

GCP-ICH Good clinical practices-International conference on harmonization

GMP Good manufacturing practices

IFN Interferon

IL-12 Interleukin-12

IP Institut Pasteur, Paris

LR Leishmaniasis recidivans

LST Leishmanin skin test

ML Mucosal- or mucocutaneous leishmaniasis

μM Micro-mole

ODN Oligodeoxynuceotide

PCR Polymerace chain reaction

PKDL Post kala azar dermal leishmaniasis

QT interval of time from the start of the Q wave to the end of the T wave in the heart's electrical cycle.

R&D Research and development

Sb^+5 ^Pentavalent antimony

SGV Second generation vaccine (defined vaccine)

T h Thymus derived helper cell

TDR The Unicef-UNDP-WHO-World Bank special program on research and training in tropical diseases

TLR Toll like receptor

TNF Tumor necrosis factor

TPP Target product profile

USAMRMC, US Army Medical Research and Materiel Command

WHO World Health Organization

WRAIR Walter Reed Army Institute of Research

ZCL Zoonotic cutaneous leishmaniasis (here referred to CL caused by *L. major*)

## Supplementary Material

Additional File 1 Product Profile for CL treatment. What characteristics should an ideal product have for treatment of CL and what is the minimum requirement for a product to be introduced for control of CL, with emphasis first on ACL and secondly of CL latin America caused by L. braziliensis. Click here for file
